# Validation-verification of a highly effective, practical human testicular tissue in vitro culture-cryopreservation procedure aimed to optimize pre-freeze and post-thaw motility

**DOI:** 10.1007/s10815-016-0659-7

**Published:** 2016-02-04

**Authors:** M. C. Schiewe, C. Rothman, A. Spitz, P. E. Werthman, S. I. Zeitlin, R. E. Anderson

**Affiliations:** Ovagen Fertility/Southern California Institute for Reproductive Sciences (SCIRS), Newport Beach, CA USA; California Cryobank (CCB), Los Angeles, CA USA; Department of Urology, University of California, Irvine (UCI), CA USA; Center for Male Reproduction and Vasectomy Reversal, Los Angeles, CA USA; Department of Urology, University of California, Los Angeles (UCLA), CA USA; Southern California Center for Reproductive Medicine (SCCRM), Newport Beach, CA USA

**Keywords:** TESE, Testicular biopsy, In vitro culture (IVC), Sperm cryopreservation, Motility

## Abstract

**Purpose:**

The aim of our paper was to validate a testicular biopsy procedure that simplifies handling, processing, and cryopreservation, while at the same time optimizes sperm motility before freezing and after thawing.

**Methods:**

Two prospective studies were conducted to verify, optimize, and understand the virtues of pre-freeze testicular tissue IVC at different temperatures (21, 30, or 37 °C). Testicular tissue was obtained from clinical specimens designated for whole tissue cryopreservation (i.e., intact mass of tubules) and/or for fresh use in IVF-ICSI cycles. Whole testicular biopsy pieces (1–3 mm^3^) were diluted in glycerol containing freeze solutions, slow cooled to 4 °C and then rapidly frozen in LN_2_ vapor. Fresh and post-thaw testicular biopsy tissue were evaluated for changes in the quantity (%) and pattern of motility (I–IV: twitching to rapid progression, respectively) over a 1 week duration. The clinical effectiveness of IVC-cryopreserved whole testicular biopsy tissue was also validated analyzing fresh embryo transfers.

**Results:**

More reliable recovery of motile testicular sperm was achieved using whole tissue freeze preservation combined with IVC (24–96 h) post-acquisition at an incubation temperature of 30 °C compared to ambient temperature (21 °C) or 37 °C. Up to 85 % of the pre-freeze motility was conserved post-thaw (+3 h) for easy ICSI selection. Sperm longevity was optimized to fresh tissue levels by implementing testicular biopsy sucrose dilution post-thaw. Favorable clinical outcomes were proven using frozen-thawed testicular biopsy sperm for ICSI.

**Conclusions:**

By employing minimal tissue manipulation, integrating pre-freeze IVC processing at 30 °C and the freezing of whole testicular biopsy tissue, we have reduced the labor and improved the efficacy of processing testicular tissue for freeze-preservation and subsequent ICSI use.

## Introduction

The use of human testicular sperm as a male infertility treatment was enabled by the development of ICSI over 2 decades ago [1–4]. However, the ability to process, evaluate, and properly use surgically acquired testicular sperm involves a steep and challenging learning curve [5–7]. Verheyen and coworkers [8] evaluated four different mechanical methods (control shredding, fine mincing, vortexing, and crushing) to dissociate testicular sperm from seminiferous tubules. There was little difference in the amount and quality of sperm dispersed by shredding or mincing before Percoll density gradient separation. However, more free sperm were isolated post-Percoll washing in the fine minced group, but not without a significant loss in total recovery of sperm. Since the quantity of sperm found in a testicular biopsy may be limited, fine needle or glass slide shredding is a common mechanical method for tissue dispersion used by embryologists [9]. Alternatively, the compression squeezing of individual tubules with a bent, smooth needle surface can be performed to push cellular contents into the culture medium [10, 11]. Many urologists however, have adopted the fine tissue mincing technique that employs dissecting scissors to generate small tubular segments and free spermatozoa in solution [8, 12, 13].

**Fig. 1:**
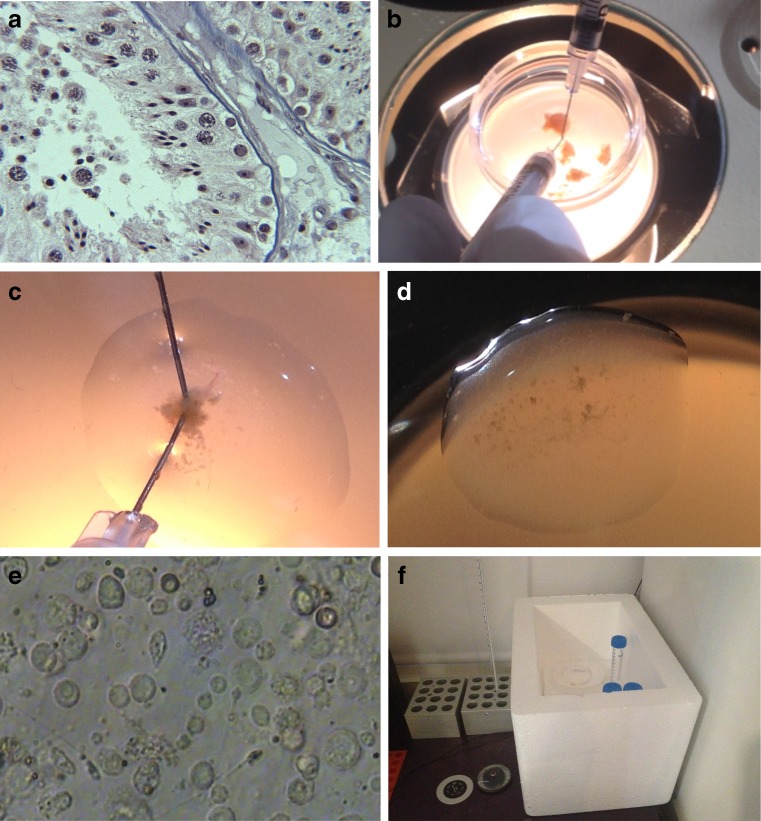
In panel **a**, a tricolor-stained histograph (×200 magnification; courtesy of Dr. Charles Sims, M.D.) of thin-sectioned testicular tissue exhibits normal spermatogenesis, from spermatogonia to sperm release in the tubule lumen. Round cell spermatid transition to sperm formation is clearly evident (**a**). The tissue mass is composed of seminiferous tubules which can be easily dissected into smaller masses (**b**) for subsequent evaluation or cryopreservation. Using fine needles to shred the tubules of a small testis mass (**c**) in a droplet of medium under oil, the dispersed cellular contents of the tissue are allowed to settle and equilibrate (**d**) before evaluation under inverted microscopy (**e**, hpf = ×400 magnification). This panel depicts normal spermatogenesis, as revealed by a variable size cell population and the presence of >5 sperm/hpf. A styrofoam box containing tube racks (**f**) is used as a testicular biopsy incubator (when covered), attaining an optimal temperature of 30 °C to culture testicular sperm, while resting on a 37 °C slide warmer surface

Independent of the method used for cellular dispersion, a fundamental problem is the time and expertise needed to sift through a three dimensional mix of testicular cells and tissue to find sperm that is generally immotile. This is complicated by the fact that the normal cellular architecture of the testis (Fig. 1a) is absent in cell suspension preparations. Medical illustrations that identify individual spermatogenic cell types (e.g., Atlas of Human Spermatogenesis) have helped aid the learning process to some degree, but has not lowered the overall expertise levels needed for full laboratory proficiency in germ cell identification.

Other issues can add to the complexity of identifying sperm in testicular biopsy samples. Often, minced testicular biopsy tissue dispersions are contaminated with high concentrations of red blood cells, making it more difficult to characterize cells and observe free floating, non-motile sperm. Erythrocyte lysing buffers are helpful in such cases in isolating motile and non-motile sperm [14]. Yet, RBC contamination can be minimized by generous irrigation during surgery and rinsing tubules well before dispersion. In cases of non-obstructive azoospermia where sperm identification is extremely difficult, enzymatic digestion of tissue has been applied to free sperm from tissue masses, allowing up to 24 % additional detection of sperm compared to non-digested preparations [15, 16]. The use of a mortar and pestal to grind tissue has also been described as a way to free sperm from host tissue [17]. Many strategies have been developed for identifying and processing testicular sperm. However, little effort has been made to implement practical measures that optimize cryopreservation outcomes, while reducing the labor demand on technicians. Instead, most laboratories thoroughly process and freeze testicular tissue on the same day of the testicular biopsy, without regard to the additional processing and post-thaw labor time that will be needed to find viable sperm for subsequent ICSI use.

**Fig. 2:**
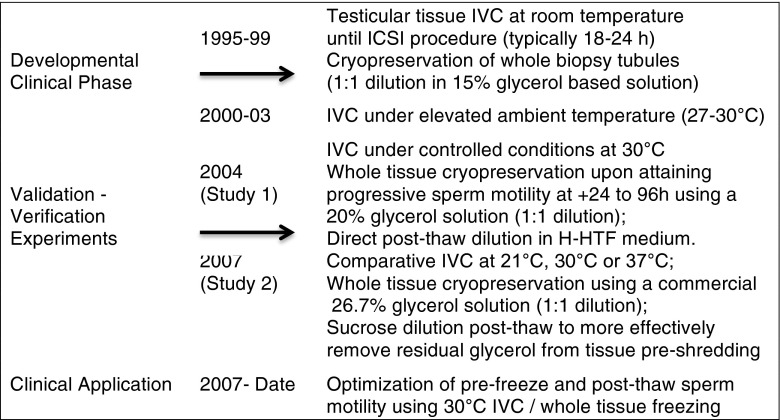
Experimental design schematic reveals the developmental phase of adopting whole testicular tissue cryopreservation and observing improvements in IVC motility characteristics associated with a change of laboratory environments/elevated ambient temperatures. In 2004, a verification study (study 1) was conducted to evaluate the beneficial effect of controlling the IVC temperature at 30 °C with daily repeated measures of motility overtime (1 week) pre-freeze and post-thaw. Subsequently, in 2007, a prospective apriori study of three different temperature treatments (21, 30, 37 °C) with repeated measures of motility was performed to validate the optimum effect of an intermediate temperature on the promotion of progressive sperm motility of longevity. Furthermore, due to the suspected negative effect of residual glycerol in post-thawed testicular tissue sperm longevity in study 1, all freeze-thawed testicular tissue in study 2 was exposed to a 0.5 M sucrose solution prior to dilution in H-HTF medium. In addition, the progressive use of higher glycerol solution concentrations was to insure effective permeation of cryoprotectant into tissue, as well as adopting a practical commercial product (shifting from a 20 % glycerol to 26.7 % glycerol solution in study 1 and 2, respectively). Finally, no randomization was performed as all repeated measures (study 1 and 2) and IVC treatments were equally applied to each patient specimen. The applications of our optimized minimal manipulation treatments (i.e., whole tissue IVC/cryopreservation) over the past 7 years have validated and verified its clinical effectiveness

In our early experience with the cryopreservation of testicular sperm, we found an interesting difference in the post-thaw viability of different morphological sperm types based on vitality stains. Maturing sperm possessing a cellular remnant encapsulating their head (i.e., late spermatid) preferentially survived freezing with significantly higher vitality (74 %) compared to free-floating normal (19 %) or abnormal sperm (31 %, bent necks/midpieces with residual cytoplasmic droplets) unassociated with tissue [18]. Interestingly, cryosurvival appeared to be associated with residual cellular protection to the centrosome region. Thus, we justified the adoption of whole tissue cryopreservation as a means to optimize cellular co-survival of testicular spermatozoa (Fig. 2). We learned that the in-vitro culturing (IVC) of testicular biopsy tissue at ambient temperature over time (+24 to +96 h) would typically enhance sperm motility pre-freeze, which benefited post-thaw motility recovery. Furthermore, under changing laboratory environmental conditions (i.e., temperature), we noticed an improvement in testicular biopsy sperm motility and longevity associated with elevated temperatures from 21 to 27–30 °C (Fig. 2), as was documented by others [19].

We have shown that ICSI of motile frozen-thawed testicular biopsy sperm yields comparable fertilization rates to fresh controls using motile or non-motile sperm (Table 1). Whereas, frozen-thawed sperm which were not distinctly motile produced fewer (*p* < 0.05; Table 1) 2PN zygotes, unless sperm encapsulated in a cellular remnant were preferentially selected. Resulting embryos by sperm type (fresh or frozen) exhibited no difference in pregnancy outcomes (Table 1). Overall, similar live births occurred using fresh (47 %) or frozen-thawed testicular biopsy sperm (50 %), which is in agreement with other reports [9, 20, 21].

**Table 1:** Clinical outcomes of IVF/ICSI cases using testicular biopsy sperm at the CFA and SIFT clinics between 1996 and 1999

TBx sperm	# Pts	# Eggs ICSI	# 2PN (%)	# CP (%)	# LB (%)	# Babies Born
Fresh	15	164	115 (70)	7 [47]	7 [47]	11
Non-motile sperm^b^	[6]	56	37 (66)	3 (50)	3 (50)	4
Type ≥II sperm	[9]	108	78 (72)	4 [44]	4 [44]	7^d^
Frozen-thawed	10	120	67 (56)	6 (60)	5 (50)	8
Non-motile sperm^b^	^c^	26	7 [27]^a^	1 (100)	1 (100)	1
Non-motile L.Sptd	[4]	26	15 (58)	2 (67)	2 (67)	3
Type ≥II sperm	[6]	66	45 (68)	3 (50)	2 [33]	4

IVF/ICSI cases excluding women ≥38 years old, *CP* clinical pregnancies, *LB* Live births

^a^Column values are different (*p* < 0.05) than all other values

^b^Micropipette “touch-test” as applied to select potentially viable spermatozoa

^c^Eggs were split evenly between sperm types in this patient group who had few, if any, twitching sperm by a “touch-response” test. The remaining sperm injected were non-motile, including the 50 % sperm encapsulated in cellular reminant, i.e., late spermatid (L Sptd) group

^d^A set of triplets was conceived using motile sperm +96 h post-TBx (1996)

Given these issues and the fact that testicular sperm retrieval procedures are more popular than ever [22, 23], it is surprising that after 20 years of practice, laboratories still struggle to find testicular tissue preparation regimens that minimize labor and effort, while optimizing sperm isolation for ICSI. The aim of this paper was to highlight the evolution of an effective, practical laboratory approach to testicular biopsy handling and processing which not only improves pre-freeze and post-thaw motility, but also minimizes the procedural time and efforts needed to optimize IVF laboratory application.

## Materials and methods

### Evaluation of testicular biopsy tissue in the operating room

Human testicular biopsy tissue was acquired from both non-obstructive azoospermic (NOA) and obstructive azoospermic (OA) patients via open surgical procedures [5, 24, 25]. Seminiferous tubular masses (i.e., testicular biopsy) were excised and placed into a small petri dish (35 mm; Fig. 1b) containing 3 ml HTF medium with HEPES buffer and 5 % HSA (H-HTF^+^; Irvine Scientific, Santa Ana, CA). By gross examination, testicular biopsies were then manipulated with sterile needles (19–28 ga) attached to 1 cc tuberculin syringes to coarsely dissect tissue. A small tissue sample (1–3 mm^3^) was teased away from the whole testicular biopsy (Fig. 1b) and placed into 1 of 4 100–150 μl droplets of H-HTF^+^ medium in a large Falcon petri dish (100 mm × 15 mm; Fig. 1d) for preliminary evaluation. Each droplet was flattened and covered with 17 ml of light mineral oil. While under oil, the representative tissue sample was shredded between the two needles (Fig. 1c) to release the cellular contents and enable spermatozoa identification using an inverted microscope (×400 magnification). If the presence of sperm was confirmed, surgical procedures were terminated; otherwise, additional testicular tissue was systematically sampled from one or both testicles. Typically, if the identification of more than a single sperm was not achieved within 10 min, multiple biopsies were taken to fully assess them later in the ART Lab. Testicular biopsy containing petri dishes was placed into a covered 30 × 20 × 20cm styrofoam box (3 cm wall thickness; Fig. 1f) and returned to the laboratory for further evaluation and processing.

### Testicular biopsy processing and assessment

Detailed processing and further evaluation of the testicular biopsy was continued in the laboratory in two stages: (1) complete processing and assessment of the initial representative sample (Fig. 1b, e) and (2) separation of whole testicular biopsy pieces for subsequent cryopreservation (Fig. 1b). Using stereomicroscopy, the representative testicular biopsy sample initially assessed in the OR (classified as a “fresh TEST sample”) was more thoroughly dissected between needles to achieve complete cellular dispersion (Fig. 1c, e) and the residual tissue moved to an unused H-HTF^+^ droplet under oil. The latter large dish was then placed on a 37 °C surface before reevaluating the TEST sample 1–3 h later. While equilibrating the shredded testicular biopsy TEST sample, the primary testicular biopsy mass(es) were subdivided by needle dissection (Fig. 1b) into a minimum of six testicular biopsy pieces (4+ whole testicular biopsy pieces for cryopreservation, a small TEST whole testicular biopsy freeze sample; as well as the initial fresh shredded TEST sample; Fig. 1d, e). The separate whole testicular biopsy pieces (1–3 mm^3^) were aseptically handled and placed into labeled, sterile individual cryovials containing 0.25 ml H-HTF^+^ and maintained in the warmed styrofoam box/racks for IVC (Fig. 1f) until the time point at which progressive motility was confirmed in the pre-freeze fresh TEST sample (typically 48–72 h). Sperm longevity was evaluated daily for up to 1 week (+168 h post-testicular biopsy). Testicular tissue was maintained under varying ambient conditions prior to 2004. Beginning in 2004, all IVC was controlled at 30 ± 1 °C in the styrofoam box maintained on a 37 °C surface, as defined previously.

The dispersed cellular contents of the testis tissue were subjectively assessed using high power field (hpf; 400×) determinations. The following criteria were used to grade the quantity of sperm found: normal = >5 sperm/hpf (Fig. 1f), moderate = 3–5 sperm/hpf, fair = 1–2 sperm/hpf, and low = <1/hpf. Very low sperm yields characteristic of difficult NOA cases was further defined as 1 sperm/number of hpf’s or perhaps as the total number of sperm found in the sample. Within 3 h post-receipt of testis tissue (time 0), a baseline examination of specimen quality was performed. In addition to an assessment of sperm quantity as described above, total percent motility was evaluated on at least 100 sperm. Sperm motility was assessed by a classification index: type: I = twitching, II = non-progressive, undulating flagellar motion, III = slow progressive motion, and IV = rapid movement. Motility was assessed daily in TEST samples of incubated testicular biopsy specimens.

### Experimental design

Our development and optimization of whole testicular biopsy IVC and cryopreservation evolved over time (Fig. 2), with prospective verification and validation studies being initiated in 2004. Although the developmental phases and experiments were performed at several facilities, all lab procedures and evaluations were conducted by a single embryologist (MCS). Duplicate evaluations were performed with the assistance of various lab staff members. In study 1, we evaluated the use of a controlled temperature (30 °C) for testicular biopsy-IVC (*n* = 12 OA men). The aim was to verify the effectiveness of testicular tissue IVC on the sperm motility of fresh and frozen-thawed specimens at 24 h intervals over a 1 week duration and to assess differences in survivability. The extended analysis was performed in conjunction with the standard assessment of TEST samples from patients consenting to whole testicular biopsy cryopreservation.

A secondary prospective, controlled validation study was conducted (*n* = 10 OA men; study 2) in conjunction to an IRB approved stem cell study [26]. Consenting patients approved the use of residual testicular biopsy tissue for experimental purposes. Each testicular biopsy was dissected into eight equal pieces. Five intact pieces of testicular biopsy were cryopreserved in separate cryovials for future use. Patient testicular biopsy tissue for cryopreservation was handled, maintained (30 °C), and processed using standard whole testicular biopsy cryopreservation procedures (as described below). The remaining testicular biopsy tissue was subdivided into one of three temperature treatment groups for each patient: room/ambient temperature (RT; 21 °C); body temperature (37 °C), or 30 °C for extended IVC. Fresh TEST tissue dishes from each patient were prepared for each different temperature condition. Comparative assessments of motility quality (type I–IV) and quantity (%) were recorded at +24, +96, and +168 h of IVC under treatment conditions. In addition, all frozen TEST samples from study 2 patients were prospectively thawed and eluted in 0.5 M sucrose solution (study 2b), as described below, in an attempt to extend sperm longevity similar to fresh tissue. Each sample was assessed daily for up to 1 week, similar to study 1.

**Fig. 3:**
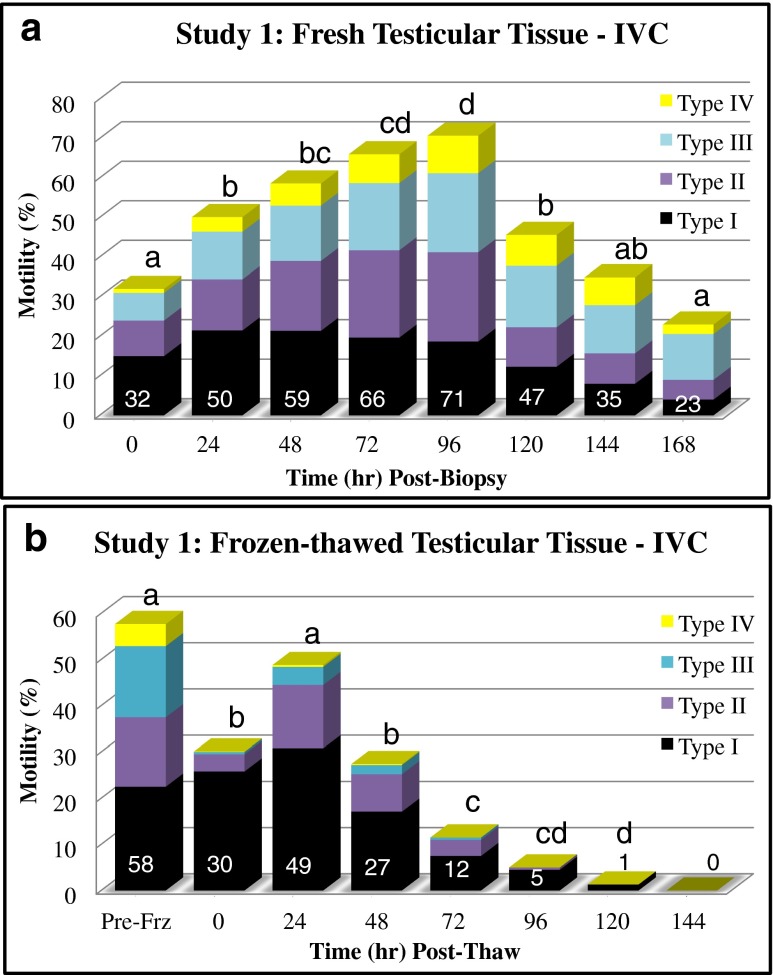
The effects of extended IVC (30 °C) on the motility of testicular sperm are graphically shown in stacked columns measuring motility types (I to IV, twitching to rapid progression, respectively; **a**). In panel **b**, post-thaw motility patterns of cryopreserved testicular biopsy sperm over time (IVC at 30 °C) are shown in stacked columns measuring the four types of motility. The total motility for each interval, in both panels, is indicated within the lower sub-column. Good post-thaw survival was recovered for cryopreserved whole testicular biopsy specimens in an actively motile state. Upon equilibration, the optimal viability of the post-thaw samples was acquired by +24 h IVC, which significantly declined at +48 and +72 h in study 1 where residual glycerol had a toxic effect on longevity. In each panel, subscript(s) atop each column denote significant differences between intervals

**Fig. 4:**
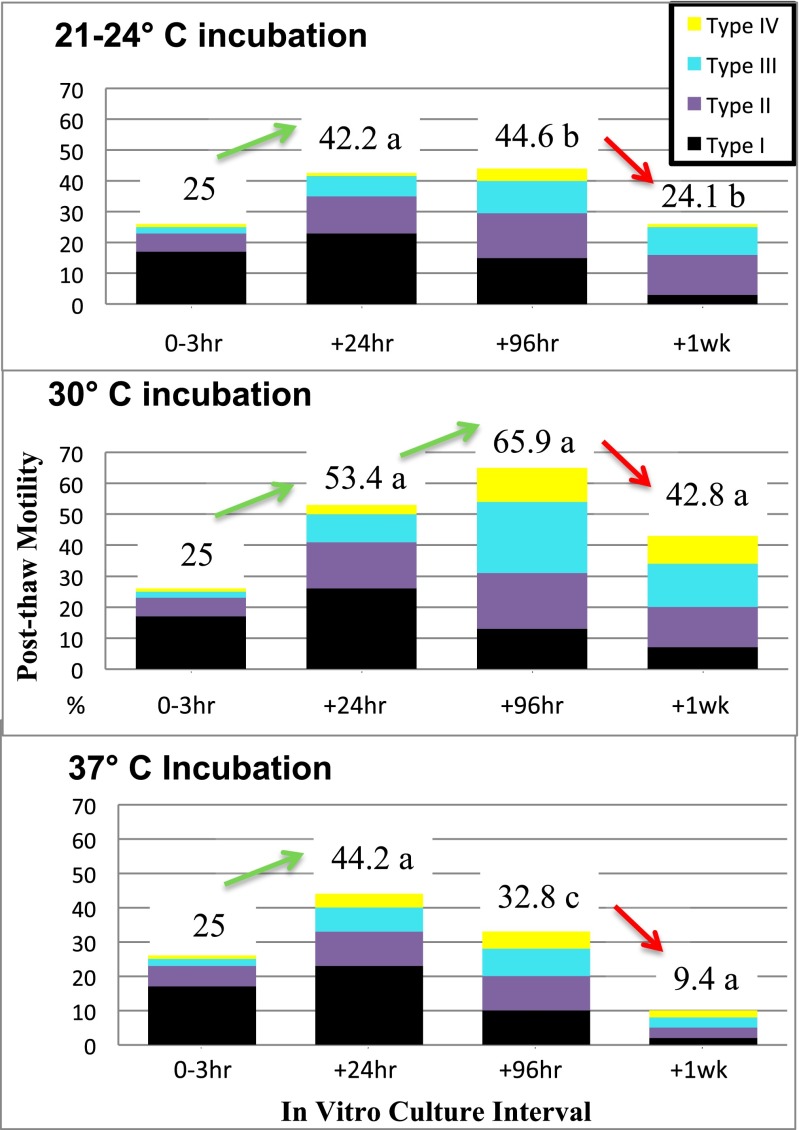
In study 2, the effect of extended IVC on the motility of testicular sperm was tested at three different incubation temperatures (21, 30, and 37 °C). The four types of motility (graded: I–IV) are graphically shown in stacked columns measuring the total motility at each of four intervals. Differences (*p* < 0.05) within each column between treatments are reflected by subscript differences adjoining the total motility value. Significant changes in motility patterns between intervals within incubation treatments are indicated by the inclusion of a directional arrow

The clinical effectiveness of IVC at an intermediate temperature of 30 °C was assessed in study 1 and study 2 for women ≤42 years old. Statistical analyses involved the use of a chi-squared test to assess differences (*p* < 0.05) in the proportion (%) of sperm viability, total motility/day, normal fertilization, clinical pregnancy, and live birth rates between treatment groups. Furthermore, treatment differences in total and progressive motility, within and between intervals (Figs. 3 and 4), were contrast by chi squared analysis.

### Testicular cryopreservation and thawing

The whole testicular biopsy tissue pieces (approximately 2 × 2 × 1 mm^3^) were cryopreserved after 30 °C IVC of 24 to +96 h post-biopsy, depending on the confirmation of type III and IV motile sperm being detected. Testis tissue was frozen in a 20 % glycerol solution (*v*/*v*) in H-HTF medium +10 % HSA that was prepared and filtered (0.22 μm) in-lab for use (2000–2007; study 1 period); or in Sperm Maintenance Medium (26.7 % glycerol, HSA-based; Irvine Scientific) after 2007 (study 2 period). Using a 5-step addition (1:1 dilution) technique over 5 to 7 min, 50 μl of freeze medium was pipette and mixed per 1–1.5 min/vial. Vials were then placed onto a labeled cane for cooling and cryostorage. A 10 to 13.4 % glycerol solution (0.5 ml final volume) was allowed to permeate the tissue for 3–18 h under refrigerated conditions (5 °C) depending on whether they were processed in the morning or afternoon, respectively. The canes were then frozen directly in LN_2_ vapor for 30 min prior to storage in LN_2_. Note, the freezing procedure was developed to be practical and effective, with no attempt being made to control the refrigeration interval or contrast changes in glycerol content.

Tissue vials were thawed initially by slow warming and equilibration at ambient temperature for 1 min. Upon outgassing of LN_2_ vapors, vials were then rapidly warmed in a 37 °C water bath for 5 min. Vial contents (i.e., whole tissue) were then either evacuated into a 4-well dish to which an equal volume of H-HTF^+^ medium was added (prior to 2007; study 1), or placed directly into a 0.5 M sucrose solution (1.78 g/warm 10 ml H-HTF; filter sterilized) at room temperature to more completely elute residual glycerol from the tissue (≥2007; study 2). With either approach, after 5 min, the testicular biopsy tissue masses were rehydrated in and rinsed twice in fresh H-HTF^+^ for 5 min intervals using sterile needles for handling at ambient temperature to decrease residual extracellular glycerol. The equilibrated tissue was then moved to fresh H-HTF^+^ droplets under oil, shred by needles and the cellular contents allowed to warm at 37 °C for 1–3 h. Upon equilibration, post-thaw assessment of motility was performed and re-evaluated daily for up to 1 week, under previously described IVC incubation conditions (30 ± 1 °C). TEST thaw results dictated whether testicular biopsy samples were thawed on the morning, or 24 h in advance, of an ICSI procedure [27].

## Results

### Study 1

Upon dilution equilibration of dispersed testicular tissue for 3 h in HTF^+^ at 37 °C sperm began exhibiting motility (32 % total), which was primarily non-progressive (24 %; Fig. 3a). However, by 24 h IVC, there was a significant rise in total motility (50 %) and progressive motility (8 to 15.7 %), which increased (*p* < 0.05) further at 72 to 96 h (24.3 and 29.5 %, respectively). A maximum average total motility of 71 % was attained at +96 h post-testicular biopsy, with a significant decline in total motility observed at +120 h, but not in progressive motility (23.3 %). Testicular sperm motility continued to decrease (*p* < 0.05) to 24 % total motility at +168 h, yet progressive motility (14 %) remained elevated to the +24 h levels.

Having typically cryopreserved the whole testicular biopsy masses at +48 h of IVC, the average pre-freeze total motility was 58 %, of which 20.2 % were considered progressive and 15 % undulating (Fig. 3b). The stress of cryopreservation reduced (*p* < 0.05) the initial post-thaw motility to 30 %, with 4.3 % being notably motile (undulating to progressive) sperm for ICSI selection. An 85 % overall recovery of viable testicular sperm was achieved by whole testicular biopsy tissue cryopreservation based on the increased total and grade II–IV motility at +24 h (49 and 18 %, respectively). The viability of these sperm significantly declined at a much faster rate than fresh tissue, reaching near zero levels at +120 h IVC (Fig. 3b).

In this study group of 12 ICSI cycles, 141 of 203 oocytes fertilized (69.5 % 2PN) with no difference observed using fresh IVC or thawed motile testicular sperm. In this mixed age group (24 to 42 years, mean = 35.4 ± 5.3 years), 6 of 12 patients initiated pregnancy. The five live births (41.7 %) produced one set of triplets and three twins, resulting in a 23.2 % implantation rate.

### Study 2

Testicular sperm motility ranged from 5 to 25 % initially (1–3 h post-testicular biopsy; primarily twitching) and increased (*p* < 0.05) in all groups at 24 h (Fig. 4). There was no difference (*p* > 0.10) in total motility at 24 h (42 to 53.4 %), but progressive motility was higher (*p* < 0.05) at 30 and 37 °C (12.7 and 11 %, respectively) compared to 21 °C (7.7 %). Significant differences (*p* < 0.05) were observed in total % motility/% progressive motility, respectively, at 96 h with treatment differences (*p < 0.05) being 30 °C (66 %*/44 %*) > 21 °C (42 %*/14 %) > 37 °C (18 %*/10 %). This statistical trend continued at 144 h with 30 °C (42 %*/23 %*) > 21 °C (24 %*/8 %) > 37 °C (9 %*/5 %). Thus, the optimization of progressive sperm motility and longevity significantly favored the 30 °C IVC treatment.

As in study 1, when patient samples were thawed after being cryopreserved at +48–72 h of 30 °C IVC, overt post-thaw motility was observed (30–40 %). Nearly 15–20 % of the thawed testicular sperm exhibited undulating to slow progressive motility (type II–III) after 3 h equilibration at 37 °C. Upon 24 h IVC at 30 °C, total and progressive motility significantly increased to 60 and 25 %, respectively, and then slowly declined to 25 and 15 %, respectively, by 1 week post-thaw. The sucrose eluted testicular biopsy tissue displayed a viability longevity curve resembling that of fresh tissue.

During this study, a viable pregnancy was achieved using a 30 °C sample 8 days post-biopsy, exceeding two previous healthy triplet pregnancies which were successful using +96 h IVC testicular biopsy specimens in 1996 and 2005. In review of 100 clinical cases performed at the SCIRS Lab, between 2011 and 2014, the routine clinical use of IVC/frozen-thawed testicular sperm has resulted in reliable, excellent clinical outcomes (Table 2) and low miscarriage results, comparable to those routinely attained with fresh ejaculated sperm.

**Table 2:** Clinical outcomes of ICSI cases using IVC/frozen-thawed whole testicular biopsy sperm at the SCIRS laboratory between 2011 and 2014

Mean female patient age	35.7 ± 4.8 SD
# of oocyte retrievals	100
# of mature oocytes injected	1356
# of 2 pronuclear zygotes	920 (67.9 %)
Total # fertilized	1023 (75 %)
# of embryo transfers-fresh embryos	60
# of positive pregnancies	35 (58.3 %)
# of clinical pregnancies	32 (53.3 %)
# of live birth outcomes	28 (47 %)

## Discussion

Over the past 2 decades, we have learned that the management of testicular biopsy/ICSI cycles does not have to be complicated. A practical solution rests in adopting a simple and effective testicular biopsy tissue processing procedure, which incorporates pre-freeze IVC motility enhancement and whole tissue cryopreservation. Fresh testicular biopsy samples incubated at 30 °C can maintain progressive motility for up to 1 week or more, and their viability can be efficiently cryopreserved as intact tissue masses. We know that testicular sperm are typically non-motile upon initial testicular biopsy analysis, and that within 3 h of IVC warming, many sperm begin to exhibit intermittent twitching or persistent undulating flagellar movement. Overtime, IVC of testicular sperm can optimize and extend progressive sperm motility. However, prolonged in vitro incubation under suboptimal conditions can reduce sperm motility and longevity, thus decreasing the efficacy of testicular biopsy tissue for cryopreservation and subsequent ICSI use. Our investigations and experience sought to address these issues.

The low overall vitality of the stained frozen-thawed testicular biopsy sperm we observed in preliminary investigations in 1996 (30 %) was similar to other reports [13, 28] and correlated to ICSI outcomes when randomly selecting non-motile frozen-thawed sperm (27 % normal fertilization, Table 1). Furthermore, we discovered that the cellular encapsulation of the sperm head/midpiece allowed the cytoplasm to insulate the centrosome from cryodamage, preserving its functionality. These early observations revealed a near 4-fold increase in the vitality of cellular protected testicular sperm compared to normal free-floating sperm, as well as a 2-fold increase in normal fertilization when injecting non-motile cells using a “touch” technique (Schiewe, unpublished data).

Interestingly, the subsequent success with IVC in study 1 determined that up to 70 % of the sperm freeze preserved as intact tubules actually sustained their viability post-thaw, based on observed post-thaw motility potential at +96 h IVC. Thus, we revealed a discrepancy with our estimated 30 % vitality assessed at 0 h post-thaw in preliminary investigations. The early efforts of Crabbé and coworkers [13] favored frozen minced tissue suspensions (9.2 % motile and 39.3 % viable) compared to whole testicular biopsy cryopreservation (4 % motile and 25.4 % viable). Their findings did not seem to support our belief that the centrosomes of normal immature sperm embedded within tissue are more likely insulated from cryodamage. However, we believe their observed differences in motility/viability were due to an experimental flaw, which did not account for the need to use higher glycerol levels to effectively permeate the whole testicular biopsy tissue pre-freeze. We have proven that whole testicular biopsy cryopreservation is a simple and highly effective approach to processing testicular biopsies. Although we did not comparatively investigate testicular biopsy cryopreservation procedures per se, we did show that testicular biopsy exposure to sucrose post-thaw effectively removed residual glycerol from tissue to extend post-thaw sperm longevity, as opposed to the poor extended survival seen in study 1 (Fig. 3B) with HTF^+^ diluted tissue. Clearly, if we are to further optimize future freeze preservation efforts for testicular sperm/biopsy tissue, there is a justified need to evaluate different cryo-additives, -protectants and procedures.

The basic standards for sperm cryopreservation have been well established [29]. They typically involve dropwise dilution of a glycerol-based cryoprotective solution over a 7 to 15 min interval at room temperature to reduce osmotic stress, yielding a 6 to 12 % glycerol solution [10, 16, 19, 29–32]. The use of egg yolk as a membrane stabilizer/protectant has been commonly replaced by human serum albumin in commercial freeze formulations. Although the primary reason for this change in the human IVF industry was the elimination of animal origin products for clinical use, it has the added benefit in freeze-thawed testicular biopsy tissue of reducing excessive “Browning movements.” In the early years of testicular biopsy freezing (1996–2000), the rupturing of cytoplasmic contents in the shredding of testicular biopsy tissue into a lipoprotein rich culture medium yielded excess kinetic energy (i.e., random movements), which complicated the ability to identify barely twitching sperm for ICSI. In turn, our whole testicular biopsy cryopreservation program evolved in the late 1990s, focusing on improving pre-freeze motility to attain more undulating to progressive motile sperm immediately post-thaw, as well as eliminating egg yolk from our freeze media.

Freshly biopsied testicular biopsy tissue has relatively low levels of undulating or progressively motile sperm within the first 3 h post-testicular biopsy. Therefore, it is not surprising that standard freezing of fresh testicular biopsy suspensions typically results in little to no sperm motility. Liu and coworkers [9] showed that the secondary culturing of testicular biopsy sperm post-thaw for ≥72 h significantly improved progressive motility from 2 to 21 %. Over the years, many programs have adopted the thawing of testicular biopsy sperm into standard practice +24 h prior to ICSI use [17, 33]. However, patient variation to the post-thaw recovery of motility varies and may show little to no improvement [9]. A hypo-osmotic swelling test (HOST) [34, 35] and the chemical addition of pentoxyfylline [36] have been used to help assess the viability or stimulate the onset of motility of testicular sperm, with the chemical treatment proving to be more reliable than HOST [37]. In addition, a mechanical touch technique has frequently been used to potentially judge the post-thaw viability of immotile testicular sperm [20]. In all these unfavorable post-thaw testicular sperm cases, embryologists’ are faced with implementing laborious, time-consuming or chemically mediated measures to search, isolate, and use potentially viable sperm for ICSI. Conversely, the simple use of motile sperm immediately post-thaw (≤3 h) will always be the preferred optimal alternative.

Our experience dictates that the more motile the testicular biopsy sperm, the better the post-thaw viability. Reliable post-thaw motility facilitates the simple and accurate selection/ isolation of viable testicular sperm for ICSI. Under static IVC conditions at 30 °C, testicular biopsy sperm acquired motility which progressively increased in the first 96 h, similar to others showing peak motility between 48 and 72 h at 32 to 37 °C IVC [9, 19, 38]. As in our study, motility significantly declines by +96 h when incubated at 37 °C. In addition, the use of motile frozen-thawed testicular biopsy sperm in our studies has produced comparable fertilization rates to other reports [31, 39], but in contrast to Aoki et al. [39], no difference in pregnancy/live birth outcomes has been seen when contrasted to fresh testicular biopsy sperm. It should be noted that while total motility begins declining after +96 h of 30 °C IVC, a proportional increase in progressive motility was seen for up to 1 week in fresh testicular biopsy tissue, as well as post-thaw specimens when sucrose elution was applied upon warming.

Clearly, the static IVC of testicular biopsy is not an optimal system in contrast to promising potential developments with microfluidic culture systems [40, 41]. One concern with extended static IVC, as well as testicular biopsy freezing, has been a proposed increase in reactive oxygen species (ROS) in solution, which could damage the DNA integrity of testicular biopsy sperm. Dalzell and coworkers [42] exhibited that the short incubation (+4 h) of fresh testicular sperm increased the DNA fragmentation index (DFI) from 11 to 22.1 %, while frozen-thawed testicular sperm experienced higher levels of change (17 to 30 %) without any further changes at +24 h (fresh or frozen). Furthermore, it was shown that slow frozen-rapid thawed testicular sperm experienced cryodamage associated with DNA fragmentation [43]. However, these studies failed to differentiate between live and dead sperm, which would explain the lack of change in DFI of fresh samples at ≥24 h IVC. Whereas our proven success with both IVC and testicular biopsy cryopreservation indirectly suggest that elevated ROS and DFI observations are non-detrimental factors, which likely are associated with non-viable sperm. It has been proposed that the use of sperm possessing elevated DFI levels for assisted reproductive technologies will have little effect on fertilization, but can significantly decrease blastocyst production and clinical pregnancy rates [44]. Yet, our clinical results over 2 decades have not reflected any impaired clinical outcomes, other than known differences reported between NOA and OA paternal sources [17, 45]. Additionally, Wyrobek and others [46] studied age effects in men and determined that motility and DFI were inversely correlated, suggesting that specimens with increased motility had decreased DFI. Emiliani and coworkers [47] further determined that testicular sperm possessing single stranded (ss)-DNA were more vulnerable to the denaturing stress of oxidation than double stranded (ds)-DNA. They further showed that the increased motility of sperm after +72 h IVC correlated to a reduction in ss-DNA and increase in ds-DNA. Overall, the accumulated evidence supports our belief that the use of overtly motile testicular sperm post-thaw minimizes concerns associated to ROS-mediated DNA fragmentation that may occur at varying levels upon IVC and post-cryopreservation.

Our experience has developed an effective management plan for testicular biopsy/ICSI cycles between urologist/oocyte retrieval schedules, operating room availability and the laboratory that does not have to be complicated. A practical solution rests in adopting a simple and effective testicular biopsy tissue-processing plan which incorporates pre-freeze IVC motility enhancement and whole tissue cryopreservation. We have learned that fresh testicular biopsy samples incubated at 30 °C can maintain progressive motility for up to 1 week or more, and their viability can be efficiently cryopreserved as intact tissue masses. Improvements in future IVC systems, including the use of microfluidics, will hold promise to enhancing motility for weeks, as well as promoting the possible in vitro maturation of diploid pre-spermatid stages (i.e., spermatocytes and spermatogonia) into functional and viable spermatozoa.
